# Neutrophil-to-Lymphocyte Ratio Predicts Mortality in Adult Renal Transplant Recipients with Severe Community-Acquired Pneumonia

**DOI:** 10.3390/pathogens9110913

**Published:** 2020-11-04

**Authors:** Yue Qiu, Ying Su, Guo-Wei Tu, Min-Jie Ju, Hong-Yu He, Zhun-Yong Gu, Cheng Yang, Zhe Luo

**Affiliations:** 1 Department of Critical Care Medicine, Zhongshan Hospital, Fudan University, Shanghai 200032, China; 20111210121@fudan.edu.cn (Y.Q.); su.ying@zs-hospital.sh.cn (Y.S.); tu.guowei@zs-hospital.sh.cn (G.-W.T.); ju.minjie@zs-hospital.sh.cn (M.-J.J.); he.hongyu@zs-hospital.sh.cn (H.-Y.H.); gu.zhunyong@zs-hospital.sh.cn (Z.-Y.G.); 2 Department of Urology, Zhongshan Hospital, Fudan University, Shanghai 200032, China; 3 Shanghai Key Laboratory of Organ Transplantation, Fudan Zhangjiang Institute, Shanghai 201203, China; 4 Department of Critical Care Medicine, Xiamen Branch, Zhongshan Hospital, Fudan University, Xiamen 361015, China

**Keywords:** neutrophil-to-lymphocyte ratio, renal transplantation, prognosis, community-acquired pneumonia, mortality

## Abstract

Mortality of renal transplant recipients with severe community-acquired pneumonia (CAP) remains high, despite advances in critical care management. There is still a lack of biomarkers for predicting prognosis of these patients. The present study aimed to investigate the association between neutrophil-to-lymphocyte ratio (NLR) and mortality in renal transplant recipients with severe CAP. A total of 111 renal transplant recipients with severe CAP admitted to the intensive care unit (ICU) were screened for eligibility between 1 January 2009 and 30 November 2018. Patient characteristics and laboratory test results at ICU admission were retrospectively collected. There were 18 non-survivors (22.2%) among 81 patients with severe CAP who were finally included. Non-survivors had a higher NLR level than survivors (26.8 vs. 12.3, *p* < 0.001). NLR had the greatest power to predict mortality as suggested by area under the curve (0.88 ± 0.04; *p* < 0.0001) compared to platelet-to-lymphocyte ratio (0.75 ± 0.06; *p* < 0.01), pneumonia severity index (0.65 ± 0.08; *p* = 0.05), CURB-65 (0.65 ± 0.08; *p* = 0.05), and neutrophil count (0.68 ± 0.07; *p* < 0.01). Multivariate logistic regression models revealed that NLR was associated with hospital and ICU mortality in renal transplant recipients with severe CAP. NLR levels were independently associated with mortality and may be a useful biomarker for predicting poor outcome in renal transplant recipients with severe CAP.

## 1. Introduction

Community-acquired pneumonia (CAP) has high morbidity and mortality, with frequent requirement for intensive care support. As a unique group of immunosuppressed patients, solid organ transplant recipients, including renal transplant recipients, are at high risk of acquiring CAP due to the routine use of immunosuppressive regimens [1,2,3]. The first-year mortality in renal transplant recipients with CAP can increase by 6- to 12-fold [4]. Notably, mortality of renal transplant recipients with severe CAP (diagnosed according to the Infectious Diseases Society of America/American Thoracic Society (IDSA/ATS) guidelines (2007) [5]) has remained high in recent years, despite advances in critical care management [6,7,8,9]. Early identification of high-risk patients would help to administer appropriate treatment and further improve clinical outcome. Many prognostic scoring systems or biomarkers aimed at stratifying risk and predicting prognosis in immunocompetent patients with CAP have been developed, such as the pneumonia severity index (PSI) [10], CURB-65 [11], and procalcitonin (PCT) [11,12,13]. However, biomarkers for predicting poor outcome in renal transplant recipients with severe CAP are still lacking.

Neutrophil-to-lymphocyte ratio (NLR), the ratio of neutrophil count to lymphocyte count, is a simple and inexpensive biomarker of systemic inflammation. NLR is reported to be associated with poor outcome and showed a better performance than PSI, CURB-65, and C-reactive protein (CRP) in predicting 30-day mortality in immunocompetent patients with CAP [14,15]. Moreover, NLR was found to be correlated with poor outcome in various diseases, such as oncology, cardio-cerebrovascular diseases, and bacteremia. Similarly, platelet-to-lymphocyte ratio (PLR) has been described as a novel potential inflammatory marker, which is associated with poor outcome in various diseases [16,17,18,19,20]. However, the role of NLR and PLR in renal transplant recipients with severe CAP remains unclear. In the present study, we investigated the association between NLR/PLR and mortality in renal transplant recipients with severe CAP.

## 2. Results

### 2.1. Patient Characteristics

**Figure 1:**
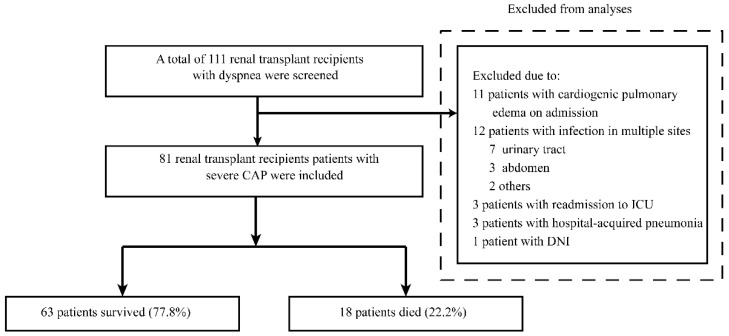
Flow diagram of our retrospective study. CAP, community-acquired pneumonia; DNI, do not intubate; ICU, intensive care unit.

A total of 111 renal transplant recipients with dyspnea admitted to the intensive care unit (ICU) between 1 January 2009 and 30 November 2018 were reviewed. Of these patients, 30 patients were excluded, including 11 with cardiogenic pulmonary edema at ICU admission, 12 with infection in multiple sites, three who were readmitted to the ICU, three with hospital-acquired pneumonia, and one with a do-not-intubate (DNI) order. Finally, 81 renal transplant recipients diagnosed to have severe CAP were included in the study (59 males (72.8%); median age, 52 [40~59] years) (Figure 1). The baseline demographics and clinical and laboratory characteristics of the 81 recipients are presented in Table 1. In the present study, 63 patients survived (77.8%) and 18 patients died during hospital stay (22.2%).

Non-survivors had a significantly lower PaO_2_/FiO_2_ ratio (*p* < 0.01) and a higher Acute Physiology and Chronic Health Evaluation II (APACHE II) score (*p* < 0.01) than survivors. However, the CURB-65 score only slightly differed between survivors and non-survivors (2 [2, 2] vs. 3 [2, 3], respectively, *p* = 0.02). PSI was comparable between both groups (110 [98, 128] vs. 126 [104, 155], respectively, *p* = 0.06). A balanced distribution was observed between survivors and non-survivors with regard to other baseline clinical characteristics, including gender, age, comorbidities prior to admission, immunosuppressive regimens, and time course before admission, except for diabetes mellitus (7.9% vs. 27.8%, respectively, *p* = 0.04).

The white blood cell (WBC) count was comparable between survivors and non-survivors (*p* = 0.07). However, lymphocytopenia was more severe in non-survivors than in survivors (0.2 [0.1, 0.3] vs. 0.5 [0.3, 0.8], *p* < 0.01), while the neutrophil level, despite in the normal range (6.1 [3.9, 10.6]), was higher in non-survivors than in survivors (8.6 [5.1, 12.8] vs. 5.7 [3.7, 9.6], *p* = 0.02). Hence, NLR was significantly higher in non-survivors than in survivors (26.8 [20.7, 99.0] vs. 12.3 [8.0, 17.6]; *p* < 0.01). The PLR was also higher in non-survivors than in survivors (705.0 [492.5, 1146.3] vs. 407.0 [242.4, 647.5]; *p* < 0.01). There were no significant differences in the levels of procalcitonin (PCT), serum creatinine (Scr), troponin T (TnT), N-terminal pro-brain natriuretic peptide (NT-proBNP), D-dimer, and C-reactive protein (CRP) between survivors and non-survivors (all *p* > 0.05).

The numbers of patients receiving invasive mechanical ventilation (IMV) and those requiring a vasopressor within 24 h after ICU admission and renal replacement treatment were higher in non-survivors (all *p* < 0.001). In addition, non-survivors had a longer ICU and hospital stay length than survivors (16 [6, 28] vs. 7 [4, 11] days, *p* = 0.01; 30 [17, 41] vs. 20 [14, 29], *p* = 0.04). 

### 2.2. Microbiological Findings

The microbiological findings are shown in Table 1. A total of 51 patients (63.0%) had positive microbiological findings during the treatment process according to noninvasive and/or invasive diagnostic tests. Bacterial infection (32.1%) was the primary cause of severe CAP in renal transplant recipients, followed by viral (25.9%) and fungal infections (25.9%). Non-survivors had a higher rate of bacterial infection than survivors (77.8% vs. 19.0%, respectively, *p* < 0.001). There were no differences in viral and fungal infections between the two groups. A total of 37% patients had unidentified pathogens. The survivors had a higher rate of undetermined etiology than non-survivors (44.4% vs. 11.1%, respectively, *p* = 0.01).

### 2.3. Value of Indicators to Predict Hospital Mortality

**Figure 2:**
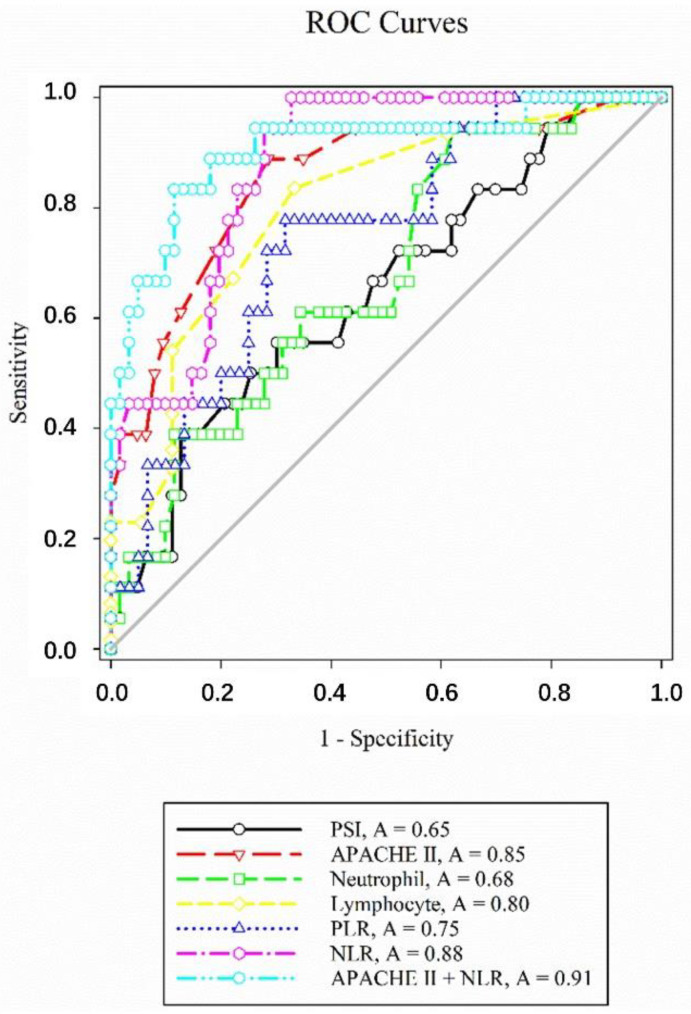
Receiver operating characteristic (ROC) curves of neutrophil-to-lymphocyte count ratio (NLR) and other indicators for predicting hospital mortality in renal transplant recipients with severe community-acquired pneumonia. APACHE II, Acute Physiology and Chronic Health Evaluation II; PLR, platelet-to-lymphocyte ratio; PSI, Pneumonia Severity Index.

Receiver operating characteristic (ROC) curves were constructed to examine the predictive performance of several indicators for hospital mortality (Figure 2). The area under the curve (AUC), optimal cutoff value, sensitivity, and specificity of each indicator are presented in Table 2. NLR showed the largest AUC of 0.88 ± 0.04, and NLR ≥ 15.25 was proposed as the optimal cutoff value, which provided a sensitivity of 100% and a specificity of 67.21% for predicting hospital mortality. PLR had an AUC of 0.75 ± 0.06, and PLR ≥ 520 was proposed as the optimal cutoff value, which provided a sensitivity of 77.78% and a specificity of 68.33% for predicting hospital mortality. Pairwise comparison of ROC curves confirmed that the AUC of NLR was significantly higher than those of PLR (*p* < 0.01), CURB-65 (*p* = 0.01), PSI (*p* = 0.01), and neutrophil count (*p* < 0.01). The AUCs of APACHE II score (*p* = 0.74) and lymphocyte count (*p* = 0.14) were identical to that of NLR. The combination of APACHE II score and NLR had an excellent prediction of hospital mortality with an AUC value of 0.91 ± 0.05 (*p* < 0.0001), which is higher than that of APACHE II score (0.85 ± 0.05; *p* < 0.0001).

Furthermore, we performed univariate and multivariate logistic regression analyses to confirm the association of each variable with mortality (Table A1 in Appendix A and Table 3 and Table 4). The multivariate logistic models showed that NLR was associated with hospital and ICU mortality in renal transplant recipients with severe CAP (model 1, adjusted odd ratio (OR), 1.07 [1.02–1.14], *p* = 0.01; model 2, adjusted OR, 1.07 [1.02–1.14], *p* = 0.01; Table 3 and Table 4, respectively), while PLR was not associated with hospital mortality and ICU mortality (models 5 and 6, all *p* > 0.05; Table 3 and Table 4). NLR was further classified into three levels: high (≥24.0), medium (16.2–24.0), and low (≤16.2), based on data distribution. The multivariate logistic models confirmed that NLR was associated with hospital and ICU mortality (models 3 and 4, all *p* for trend < 0.01; Table 3 and Table 4).

## 3. Discussion

To the best of our knowledge, this is the first study to focus on the predictive value of NLR in renal transplant recipients with severe CAP. Our study demonstrated that elevated NLR level was associated with mortality in renal transplant recipients with severe CAP. Moreover, NLR had better performance than PLR, PSI, and CURB65 score in predicting mortality. 

Inflammatory responses play an important role in the development and progression of CAP. As a simple and convenient marker of inflammatory status, NLR was found to be an independent predictor of mortality in immunocompetent patients with CAP, including children and elderly patients [14,15,21,22,23]. Moreover, NLR was recently considered as an independent biomarker in patients with coronavirus disease 2019 (COVID-19) [18,24]. Generally speaking, neutrophilia and/or lymphopenia are common immune responses during infection, which finally result in elevated NLR level. The possible mechanisms that drive neutrophilia and lymphopenia include demargination and delayed apoptosis of neutrophils as well as margination and accelerated apoptosis of lymphocytes [25,26,27,28]. 

NLR has also been reported to be correlated with a wide range of disorders. On the one hand, NLR was found to be associated with poor prognosis in various conditions, such as severe infections, systemic inflammation, acute coronary syndrome, and cancer [29,30,31,32,33,34]. On the other hand, it has been demonstrated that high NLR level was associated with renal recovery in patients with rapidly progressive glomerulonephritis [19]. These findings indicate the complex role of NLR in various diseases. To date, the role of NLR in renal transplant recipients with severe CAP remains unknown. 

Renal transplant recipients, who are usually administered immunosuppressive agents and steroids, are a subgroup of immunocompromised populations. As innate and adaptive immune responses are usually inhibited by immunosuppressive agents and chronic steroid administration, neutropenia and lymphocytopenia are common in renal transplant recipients [35]. Therefore, an inflammatory response against pneumonia in renal transplant recipients differs greatly from those in immunocompetent patients. In the present study, lymphocytopenia was common in renal transplant recipients with severe CAP, while the median neutrophil level was in the normal range. Lymphocytopenia was more severe in non-survivors than in survivors, while the neutrophil level, despite in the normal range, was higher in non-survivors than in survivors. These factors led to higher NLR in non-survivors than in survivors. 

PLR has also been highlighted as a potential new marker of systemic inflammation. Several studies have reported that PLR is linked with poor outcome in various diseases, including COVID-19, rheumatoid arthritis, glomerulonephritis, and cancer [16,17,18,19,20]. However, our results did not confirm the association between the PLR level and mortality in renal transplant recipients with severe CAP. PLR has lower AUC values in predicting hospital mortality than NLR. This may be partially explained by the comparable platelet levels between survivors and non-survivors.

Many prognostic scoring systems aimed at stratifying risk in patients with CAP have been developed, such as PSI and CURB-65. Their performances in predicting mortality have been validated in large, independent populations that included immunocompetent patients with CAP [36,37]. However, their performances in renal transplant recipients with severe CAP were unsatisfactory when compared with that of NLR. Thus, common scoring systems, such as PSI and CURB-65, may not be suitable for renal transplant patients with severe CAP. 

In the present study, bacterial infection was the primary cause of severe CAP in renal transplant recipients. Non-survivors had a higher rate of bacterial infection than survivors. Multivariate logistic regression models also confirmed that bacterial infection in these patients was associated with mortality. This implied that renal transplant recipients with CAP caused by bacteria tended to be susceptible to develop highly severe disease, resulting in poor outcome. However, it should be noted that 37% of our patients had undetermined microbiological findings, and non-survivors had a lower rate of undetermined pathogens than survivors. The possible reasons for this finding are that non-survivors had high severity of disease and tended to receive invasive diagnostic procedures to determine the etiology, such as fiberoptic bronchoscopy with bronchoalveolar lavage.

The rate of invasive mechanical ventilation was lower in survivors than in non-survivors (4.8% vs. 88.9%, respectively, *p* < 0.001). The possible reasons for this finding are that interdisciplinary approaches have been implemented in our center since 2009 to delay or avoid intubation, which include appropriate oxygen therapy, adjustment of immunosuppressive regimen and steroids, individual antibiotic therapy, and so on [8,38,39,40,41]. The overall mortality was, therefore, markedly decreased from nearly 50% prior to 2009 to 22.2% [40].

This retrospective study has several limitations. First, the study was limited by the small sample size, which might have affected the statistical power and restricted stratified analysis. Further confirmation should be obtained using a larger cohort in future studies. Second, other inflammatory indicators, such as interferon-gamma (IFN-γ) and tumor necrosis factor-alpha (TNF-α), were not measured in our study. Third, the conclusion based on kidney transplant recipients may not be generalized for all immunosuppressed patients. 

## 4. Materials and Methods

### 4.1. Study Design

The study protocol complied with the standards of the Declaration of Helsinki and current ethical guidelines and was approved by the Ethics Committee of Zhongshan Hospital, Fudan University. Renal transplant recipients with dyspnea admitted to the ICU of Zhongshan Hospital, Fudan University, between 1 January 2009 and 30 November 2018 were retrospectively reviewed.

CAP was diagnosed as pneumonia acquired outside of a hospital setting [42]. Hospital-acquired pneumonia (HAP) was defined according to previous reports [43]. Severe CAP was diagnosed according to the Infectious Diseases Society of America/American Thoracic Society (IDSA/ATS) guidelines (2007) [5]. Patients who met either one major criterion or at least three minor criteria were diagnosed to have severe CAP. The major criteria included the following: (1) septic shock in need of vasopressors and (2) respiratory failure requiring mechanical ventilation. The minor criteria included the following: (1) PaO_2_/FiO_2_ ratio < 250 mmHg; (2) respiratory rate (RR) > 30 breaths/min; (3) infiltration in multilobes; (4) confusion/disorientation; (5) uremia (blood urea nitrogen (BUN) > 20 mg/dl); (6) leukopenia (white blood cell (WBC) count = 4000/mL); (7) thrombocytopenia (platelet count < 100,000/mL); (8) hypothermia (core temperature < 36 °C); (9) hypotension requiring aggressive fluid resuscitation. Patients were excluded if they met the following criteria: (1) HAP; (2) cardiogenic pulmonary edema on admission; (3) readmission to ICU; (4) concurrent infection in other sites (urinary tract, abdomen, and so on); (5) do-not-intubate (DNI) order.

### 4.2. ICU Management and Microbiological Diagnostic Approach

A summary of standardized approaches for renal transplant recipients with severe pneumonia has been described in our previous studies [8,44]. High-resolution computed tomography (HRCT) examination was performed for diagnosis and evaluation before hospital admission and during ICU stay. 

Diagnostic tests for identifying the pathogens of severe CAP included noninvasive procedures (urine and sputum cultures) and invasive diagnostic procedures (fiberoptic bronchoscopy with bronchoalveolar lavage, blood culture, and detection of serum antibodies against cytomegalovirus (CMV), Epstein–Barr virus (EBV), *Legionella*, and *Mycoplasma*). Additionally, the 1,3-β-d glucan test (G test), galactomannan antigen detection (GM test), T cell spot test for tuberculosis (T-SPOT), and tuberculin test were performed. 

### 4.3. Data Collection

Patient demographics (baseline clinical characteristics including the Acute Physiology and Chronic Health Evaluation II (APACHE II) score, Pneumonia Severity Index (PSI), CURB-65 score, and PaO_2_/FiO_2_ ratio), laboratory data at ICU admission (including hemoglobin level, platelet count, white blood cell count, alanine aminotransferase (ALT) level, aspartate aminotransferase (AST) level, alkaline phosphatase (ALP) level, C-reactive protein (CRP) level, D-dimer level, γ-glutamyl transpeptidase (γ-GT) level, bilirubin level, troponin T (TnT) level, N-terminal pro-brain natriuretic peptide (NT-proBNP) level, serum creatinine (Scr) level, glomerular filtration rate (GFR), and procalcitonin (PCT) level) and microbiological findings were collected. In our center, complete blood count was measured using the Beckman Coulter LH-750 Hematology Analyzer (Beckman Coulter, Inc., Fullerton, CA, USA). Blood biochemical parameters were measured by an automatic biochemistry analyzer (Roche Cobas 8000 modular analyzer, Roche Diagnostics, Mannheim, Germany). NT-proBNP was measured by the Elecsys Electro-chemo luminescent assay (Cobas e 411 analyzer, Roche Diagnostics). Treatments during ICU stay, including vasopressor use within 24 h after ICU admission, renal replacement therapy, and invasive mechanical ventilation (IMV), and clinical outcomes, including length of ICU stay and hospital stay and ICU and hospital mortality, were also recorded. NLR was calculated using the following formula: NLR (%) = Neutrophil count/Lymphocyte count × 100%. 

### 4.4. Statistical Analysis

Categorical variables were compared using the chi-square test or Fisher’s exact test, while continuous variables were compared using the Mann–Whitney U test. To determine the sensitivity and specificity of variables to predict mortality, receiver operating characteristic (ROC) curves were plotted, and the area under the curve (AUC) and Youden’s index were then calculated from ROC curves. The optimal cutoff value was based on Youden’s index. The ROC curve for the combination of APACHE II and NLR for predicting hospital mortality was also plotted according to the Mackinnon and Mulligan’s weighted sum rule [45]. Pairwise comparison of ROC curves was conducted using the method of DeLong et al., 1988 [46]. Univariate logistic regression analysis was used to identify the possible risk factors for hospital mortality and ICU mortality. Variables with probability (*p*) value less than 0.1 in univariate models were introduced into the multivariate logistic models. A *p* value of less than 0.05 was considered to be statistically significant. SPSS software package, version 13.0 (SPSS Inc., Chicago, IL, USA) was used for statistical analysis of the data. 

## 5. Conclusions

NLR was independently associated with mortality in renal transplant recipients with severe CAP. NLR may be a useful prognostic marker for renal transplant recipients with severe CAP. 

## Figures and Tables

**Table 1:** Clinical characteristics of patients according to hospital mortality.

	All Patients (n = 81)	Survivors (n = 63)	Non-Survivors (n = 18)	*p* Value
**Patient demographics**				
Gender (male), n (%)	59 (72.8)	45 (71.4)	14 (77.8)	0.77
Age, Median [IQR]	52 [40, 59]	51 [37, 59]	55 [50, 62]	0.21
**Baseline clinical characteristics**				
Comorbidities				
Diabetes mellitus, n (%)	10 (12.3)	5 (7.9)	5 (27.8)	0.04
Hypertension, n (%)	61 (75.3)	47 (74.6)	14 (77.8)	1.00
Immunosuppression regimens before admission				
CsA + MMF + Pred, n (%)	32 (39.5)	25 (39.7)	7 (38.9)	1.00
TAC + MMF + Pred, n (%)	43 (53.1)	33 (52.4)	10 (55.6)	1.00
Rapa + MMF + Pred, n (%)	6 (7.4)	5 (7.9)	1 (5.6)	1.00
Acute rejection history, n (%)	13 (16.0)	9 (14.3)	4 (22.2)	0.47
APACHE II score	12 [9, 16]	11 [9, 14]	19 [15, 28]	<0.01
PSI score	114 [99, 134]	110 [98, 128]	126 [104, 155]	0.06
CURB-65 score	2 [2, 3]	2 [2, 2]	3 [2, 3]	0.02
PaO_2_/FiO_2_ ratio (mmHg)	241 [168, 370]	266 [187, 404]	176 [123, 219]	<0.01
Median time from transplantation to ICU admission, months	8 [3, 56]	8 [3, 54]	8 [3, 100]	0.95
Time from transplantation to ICU admission				0.82
The first year and after the first 30 days post-transplant, n (%)	50 (61.7)	39 (61.9)	11 (61.1)	
1–5 years post-transplant, n (%)	12 (14.8)	10 (15.9)	2 (11.1)	
>5 years post-transplant, n (%)	19 (23.5)	14 (22.2)	5 (27.8)	
Median time from fever onset to hospital admission, days	6 [3, 7]	6 [3, 7]	6 [2, 8]	0.98
Median time from fever onset to ICU admission, days	7 [4, 12]	7 [4, 11]	9 [4, 26]	0.25
**Laboratory characteristics**				
Hemoglobin (g/L)	105 [91, 123]	106 [91, 121]	103 [92, 126]	0.86
Platelet (10^9^/L)	198 [148, 230]	202 [158, 249]	173 [98, 225]	0.14
White blood cell count (10^9^/L)	7.5 [4.8, 11.7]	7.0 [4.2, 10.4]	8.9 [5.5, 13.5]	0.07
Neutrophil (10^9^/L)	6.1 [3.9, 10.6]	5.7 [3.7, 9.6]	8.6 [5.1, 12.8]	0.02
Lymphocyte (10^9^/L)	0.4 [0.2, 0.8]	0.5 [0.3, 0.8]	0.2 [0.1, 0.3]	<0.01
NLR	15.0 [8.6, 25.5]	12.3 [8.0, 17.6]	26.8 [20.7, 99.0]	<0.01
PLR	445.0 [269.4, 774.2]	407.0 [242.4, 647.5]	705.0 [492.5, 1146.3]	<0.01
ALT (U/L)	21 [11, 42]	20 [11, 37]	34 [17, 56]	0.18
AST (U/L)	26 [19, 34]	25 [18, 31]	34 [21, 46]	0.09
ALP (U/L)	69 [52.5, 102]	68.5 [53.5, 94.5]	82 [51, 117]	0.60
γ-GT (U/L)	35 [22, 62]	30 [20, 50]	65 [32, 103]	0.01
Total bilirubin (μmol/L)	6.5 [4.9, 9.7]	6.5 [4.9,9.2]	7.0 [5.0, 18.1]	0.41
Troponin T (ng/mL)	0.02 [0.01, 0.08]	0.02 [0.01,0.08]	0.03 [0.02, 0.11]	0.23
NT-proBNP (pg/mL)	607 [366, 2008]	594 [352, 1939]	647 [417, 3690]	0.43
Creatinine (μmol/L)	121 [93, 179.5]	117 [92, 170]	167 [103, 285]	0.09
Blood urea nitrogen (mmol/L)	13.2 [2.9, 47.7]	10.9 [10.3,11.5]	21.5 [18.4, 24.6]	<0.0001
GFR(CKD-EPI) (ml/min/1.73m^2^)	51.48 [35.4, 76.2]	38.00 [18.9,72.4]	51.97 [38.4, 76.7]	0.25
Procalcitonin (ng/mL)	0.16 [0.1, 0.5]	0.16 [0.1,0.3]	0.25 [0.1, 0.7]	0.64
D-dimer (mg/L)	2.45 [1.2, 5.9]	2.45 [1.2, 5.8]	2.51 [1.2, 7.2]	0.61
CRP (mg/L)	58.2 [37.0, 83.8]	56.2 [36.5, 83.3]	59.6 [40.5, 91.1]	0.35
**Microbiological identifications**				
Bacterial infection, n (%)	26 (32.1)	12 (19.0)	14 (77.8)	<0.001
Fungal infection, n (%)	21 (25.9)	17 (27.0)	4 (22.2)	0.77
*Pneumocystis jirovecii*, n (%)	10 (12.3)	10 (15.9)	0 (0)	0.11
Non-*Pneumocystis jirovecii*, n (%)	11 (13.6)	7 (11.1)	4 (22.2)	0.25
Viral infection, n (%)	21 (25.9)	14 (22.2)	7 (38.9)	0.22
Mycobacterium species, n (%)	2 (2.5)	2 (3.2)	0 (0)	1.00
Mycoplasma, n (%)	7 (8.6)	6 (9.5)	1 (5.6)	1.00
Undetermined, n (%)	30 (37.0)	28 (44.4)	2 (11.1)	0.01
**ICU management**				
Need for invasive mechanical ventilation (IMV), n (%)	19 (23.5)	3 (4.8)	16 (88.9)	<0.001
Vasopressors within 24 h after ICU admission, n (%)	15 (18.5)	3 (4.8)	12 (66.7)	<0.001
Renal replacement therapy, n (%)	16 (19.8)	6 (9.5)	10 (55.6)	<0.001
**Outcome**				
Median length of ICU stays, days	7 [4, 14]	7 [4, 11]	16 [6, 28]	0.01
Median length of hospital stays, days	21 [14, 31]	20 [14, 29]	30 [17, 41]	0.04
ICU mortality, n (%)	16 (19.8)	0 (0)	16 (88.9)	<0.01
Hospital mortality, n (%)	18 (22.2)	0 (0)	18 (100)	<0.01

The baseline immunosuppressive regimens included cyclosporine A (CsA), tacrolimus (TAC), mycophenolate mofetil (MMF), rapamycin (Rapa), and prednisone (Pred). APACHE II, Acute Physiology and Chronic Health Evaluation II; ALT, alanine aminotransferase; AST, aspartate aminotransferase; ALP, Alkaline phosphatase; CRP, C-reactive protein; GFR, glomerular filtration rate; γ-GT, γ-glutamyl transpeptidase; NLR, neutrophil-to-lymphocyte ratio; PLR, platelet-to-lymphocyte ratio; PSI, Pneumonia Severity Index.

**Table 2:** Performance of variables in predicting hospital mortality.

	AUC ROC	95% CI	*p*	Cut-Off	Sensitivity (%)	Specificity (%)
APACHE II score	0.85 ± 0.05	0.76–0.92	<0.0001	13	88.89	71.43
CURB-65	0.65 ± 0.08	0.54–0.76	0.05	2	50	80.95
PSI	0.65 ± 0.08	0.54–0.75	0.05	14	38.89	87.3
Lymphocyte	0.80 ± 0.06	0.70–0.88	<0.0001	0.2	66.67	83.61
Neutrophil	0.68 ± 0.07	0.56–0.78	0.01	4.3	94.44	37.7
NLR	0.88 ± 0.04	0.78–0.94	<0.0001	15.25	100	67.21
PLR	0.75 ± 0.06	0.62–0.87	<0.01	520	77.78	68.33
APACHE II score + NLR	0.91 ± 0.05	0.82–0.99	<0.0001	-	-	-

AUC ROC, area under the receiver operating characteristic curve; CI, confidence interval; APACHE II, Acute Physiology and Chronic Health Evaluation II; NLR, neutrophil-to-lymphocyte ratio; PLR, platelet-to-lymphocyte ratio; PSI, Pneumonia Severity Index.

**Table 3:** Independent predictors of hospital mortality according to multivariate logistic regression analysis.

Variables	Odds Ratio (95% CI)	*p* Value
**Model 1**		
APACHE II score at ICU admission	1.22 (1.02–1.46)	0.03
History of diabetes mellitus	1.81 (0.20–16.49)	0.60
NLR	1.07 (1.02–1.14)	0.01
Platelet	1.00 (0.99–1.01)	0.74
Procalcitonin	1.99 (0.11–36.42)	0.64
**Model 2**		
NLR level		***p* for trend**
Low (≤16.2)	Reference	0.005
Medium (16.2–24.0)	24.11 (1.81, 320.67)	
High (≥24.0)	30.13 (2.62, 347.06)	
**Model 3**		
APACHE II score at ICU admission	1.28 (1.07, 1.54)	0.007
History of diabetes mellitus	2.13 (0.23–19.49)	0.50
Neutrophil count	1.17 (1.00–1.37)	0.06
PLR	1.001 (1.00–1.003)	0.10
Procalcitonin	0.52 (0.03–9.01)	0.66
**Model 4**		
APACHE II score at ICU admission	1.19 (0.91–1.55)	0.20
History of diabetes mellitus	0.30 (0.01–7.36)	0.46
NLR	1.08 (1.00–1.16)	0.045
Platelet	1.001 (0.99–1.01)	0.87
Procalcitonin	31.08 (0.20–4935.41)	0.18
Bacterial infection	76.0 (3.41–1694.15)	0.006

Model 1 chose hospital mortality as the dependent variable and was adjusted for APACHE II score at ICU admission, history of diabetes mellitus, NLR, platelet count, and procalcitonin level. Model 2 chose hospital mortality as the dependent variable and was adjusted for APACHE II score at ICU admission, history of diabetes mellitus, NLR (as a categorical variable), platelet count, and procalcitonin level. Model 3 chose hospital mortality as the dependent variable and was adjusted for APACHE II score at ICU admission, history of diabetes mellitus, neutrophil count, PLR, and procalcitonin level. Model 4 chose hospital mortality as the dependent variable and was adjusted for APACHE II score at ICU admission, history of diabetes mellitus, NLR, platelet count, procalcitonin level, and bacterial infection. As the APACHE II score has repeated items of PaO_2_/FiO_2_ ratio and white blood cell count, APACHE II score was added only to the multivariate analysis. APACHE II, Acute Physiology and Chronic Health Evaluation II; CI, confidence interval; NLR, neutrophil-to-lymphocyte ratio; PLR, platelet-to-lymphocyte ratio.

**Table 4:** Independent predictors of ICU mortality according to multivariate logistic regression analysis.

Variables	Odds Ratio (95% CI)	*p* Value
**Model 1**		
APACHE II score at ICU admission	1.16 (0.98–1.38)	0.08
NLR	1.07 (1.02–1.14)	0.01
Platelet	1.00 (0.99–1.01)	0.46
Procalcitonin	1.95 (0.10–39.65)	0.66
**Model 2**		
NLR level		***p* for trend**
Low (≤16.2)	Reference	0.009
Medium (16.2–24.0)	15.41 (1.20, 197.32)	
High (≥24.0)	20.83 (2.06, 210.95)	
**Model 3**		
APACHE II score at ICU admission (per point)	1.25 (1.05, 1.49)	0.01
Neutrophil count	1.16 (0.99–1.36)	0.07
PLR	1.001 (1.00–1.003)	0.08
Procalcitonin	0.53 (0.04–7.14)	0.64
**Model 4**		
APACHE II score at ICU admission	1.11 (0.91–1.36)	0.32
NLR	1.07 (1.002–1.14)	0.04
Platelet	0.997 (0.99–1.01)	0.66
Procalcitonin	3.07 (0.06–170.6)	0.58
Bacterial infection	19.16 (1.96–187.3)	0.01

Model 1 chose ICU mortality as the dependent variable and was adjusted for APACHE II score at ICU admission, NLR, platelet count, and procalcitonin level. Model 2 chose ICU mortality as the dependent variable and was adjusted for APACHE II score at ICU admission, NLR (as a categorical variable), platelet count, and procalcitonin. Model 3 chose ICU mortality as the dependent variable and was adjusted for APACHE II score at ICU admission, neutrophil count, PLR, and procalcitonin level. Model 4 chose ICU mortality as the dependent variable and was adjusted for APACHE II score at ICU admission, NLR, platelet count, procalcitonin level, and bacterial infection. As the APACHE II score has repeated items of PaO_2_/FiO_2_ ratio and white blood cell count, APACHE II score was added only to the multivariate analysis. APACHE II, Acute Physiology and Chronic Health Evaluation II; CI, confidence interval; NLR, neutrophil-to-lymphocyte ratio; PLR, platelet-to-lymphocyte ratio.

## Appendix A

**Table A1:** Predictors of hospital mortality and ICU mortality according to univariate logistic regression analysis.

	Hospital Mortality		ICU Mortality	
Variables	Odds Ratio (95% CI)	*p* Value	Odds Ratio (95% CI)	*p* Value
Age	1.03 (0.98–1.08)	0.18	1.03 (0.98–1.07)	0.258
Gender	0.71 (0.21–2.6)	0.59	0.56 (0.14–2.19)	0.40
Diabetes mellitus	4.46 (1.13–17.70)	0.03	1.91 (0.44–8.40)	0.39
Hypertension	1.19 (0.34–4.15)	0.78	0.98 (0.28–3.47)	0.98
Acute rejection history	1.71 (0.46–6.39)	0.42	2.07 (0.55–7.86)	0.28
APACHE II score (per point)	1.30 (1.13–1.49)	0.000	1.27 (1.11–1.44)	0.000
PaO_2_/FiO_2_ ratio (per 10 mmHg)	0.88 (0.81–0.96)	0.002	0.90 (0.83–0.97)	0.006
PSI score (per 10 point)	1.23 (1.01–1.49)	0.04	1.24 (1.01–1.51)	0.04
CURB-65 (per point)	2.83 (1.20–6.65)	0.02	3.31 (1.34–8.21)	0.01
White blood cell count (per 10^9^/L)	1.10 (1.00–1.21)	0.05	1.10 (1.00–1.22)	0.05
Neutrophil (per 10^9^/L)	1.13 (1.01–1.25)	0.03	1.13 (1.01–1.25)	0.03
Lymphocyte (per 10^9^/L)	0.02 (0.001–0.29)	0.006	0.008 (0.00–0.26)	0.006
NLR	1.07 (1.03–1.12)	0.002	1.08 (1.03–1.13)	0.002
PLT (per 10^9^/L)	0.99 (0.99–1.00)	0.07	0.99 (0.98–1.00)	0.03
PLR	1.002 (1.00–1.003)	0.006	1.002 (1.001–1.003)	0.005
Total bilirubin	1.05 (0.98–1.12)	0.18	1.05 (0.98–1.13)	0.14
ALT	1.01 (1.00–1.03)	0.13	1.01 (1.00–1.03)	0.12
AST	1.03 (1.00–1.06)	0.10	1.02 (1.00–1.05)	0.11
ALP	1.003 (0.99–1.02)	0.72	0.997 (0.98–1.01)	0.75
γ-GT	1.001 (1.00–1.01)	0.67	0.999 (0.994–1.005)	0.85
GFR	1.002 (0.99–1.02)	0.70	0.99 (0.97–1.01)	0.27
Troponin T	0.95 (0.01–91.02)	0.98	1.57 (0.02–135.54)	0.84
Ln NT–proBNP	1.32 (0.80–2.17)	0.28	1.45 (0.85–2.46)	0.17
Procalcitonin	4.32 (1.21–15.47)	0.02	3.95 (1.17–13.29)	0.03
CRP	1.01 (0.99–1.03)	0.22	1.01 (1.00–1.03)	0.11
D–Dimer	1.08 (0.94–1.23)	0.30	1.08 (0.94–1.25)	0.27
Bacterial infection	14.88 (4.15–53.33)	<0.01	10.93 (3.05–39.18)	<0.01
Fungal infection	2.29 (0.59–8.91)	0.23	2.76 (0.70–10.94)	0.15
Viral infection	2.23 (0.73–6.82)	0.16	1.39 (0.42–4.61)	0.59
Mycoplasma	0.56 (0.06–4.97)	0.60	0.66 (0.07–5.87)	0.71

APACHE II, Acute Physiology and Chronic Health Evaluation II; ALT, alanine aminotransferase; AST, aspartate aminotransferase; ALP, Alkaline phosphatase; CRP, C-reactive protein; GFR, glomerular filtration rate; γ-GT, γ-glutamyl transpeptidase; NLR, neutrophil-to-lymphocyte ratio; PLR, platelet-to-lymphocyte ratio; PSI, Pneumonia Severity Index.
